# Anterior cingulate cortex and its projections to the ventral tegmental area regulate opioid withdrawal, the formation of opioid context associations and context-induced drug seeking

**DOI:** 10.3389/fnins.2022.972658

**Published:** 2022-08-05

**Authors:** Greer McKendrick, Dillon S. McDevitt, Peter Shafeek, Adam Cottrill, Nicholas M. Graziane

**Affiliations:** ^1^Neuroscience Program, Penn State College of Medicine, Hershey, PA, United States; ^2^Department of Anesthesiology and Perioperative Medicine, Penn State College of Medicine, Hershey, PA, United States; ^3^Medicine Program, Penn State College of Medicine, Hershey, PA, United States; ^4^Departments of Anesthesiology and Perioperative Medicine and Pharmacology, Penn State College of Medicine, Hershey, PA, United States

**Keywords:** anterior cingulate cortex, ventral tegmental area, oxycodone, self-administration, CPP

## Abstract

Clinical evidence suggests that there are correlations between activity within the anterior cingulate cortex (ACC) following re-exposure to drug-associated contexts and drug craving. However, there are limited data contributing to our understanding of ACC function at the cellular level during re-exposure to drug-context associations as well as whether the ACC is directly related to context-induced drug seeking. Here, we addressed this issue by employing our novel behavioral procedure capable of measuring the formation of drug-context associations as well as context-induced drug-seeking behavior in male mice (8–12 weeks of age) that orally self-administered oxycodone. We found that mice escalated oxycodone intake during the long-access training sessions and that conditioning with oxycodone was sufficient to evoke conditioned place preference (CPP) and drug-seeking behaviors. Additionally, we found that thick-tufted, but not thin-tufted pyramidal neurons (PyNs) in the ACC as well as ventral tegmental area (VTA)-projecting ACC neurons had increased intrinsic membrane excitability in mice that self-administered oxycodone compared to controls. Moreover, we found that global inhibition of the ACC or inhibition of VTA-projecting ACC neurons was sufficient to significantly reduce oxycodone-induced CPP, drug seeking, and spontaneous opioid withdrawal. These results demonstrate a direct role of ACC activity in mediating context-induced opioid seeking among other behaviors, including withdrawal, that are associated with the DSM-V criteria of opioid use disorder.

## Introduction

Among the estimated 2.5 million people with opioid use disorder (OUD), it is estimated that 600,000 are abusing heroin, while 1.9 million are using prescription opioids (O’Donnell et al., 2017). Additionally, subjects diagnosed with OUD relapse within the first week of sobriety (59%) and are most likely to relapse within the first month after being discharged from a detox program (80%) (Smyth et al., 2010). Currently, there are no treatment options for context-induced relapse, which is relapse caused by re-exposure to contexts previously paired with drug intake (O’Brien and Ternes, 1986). Medication assisted treatments (e.g., methadone and buprenorphine) have shown promise in reducing mortality, relapse, and prolonging abstinence (Mattick et al., 2014; Connery, 2015; Sordo et al., 2017). However, the retention rates for MAT are typically less than a year for a disorder that lasts a lifetime (Timko et al., 2016). Because of this, alternative treatment options are required, but discovery of such novel therapeutics depends upon a more complete understanding of the underlying mechanism involved in context-induced relapse to prescription opioids.

Clinical studies using blood oxygen-level-dependent (BOLD) functional magnetic resonance imaging (fMRI) have shown that, following exposure to drug-associated context/cues, the ACC, the frontal part of the cingulate cortex, is reliably activated in patients diagnosed with psychostimulant, nicotine, cannabis, or OUDs (Maas et al., 1998; Sell et al., 1999; Garavan et al., 2000; Wexler et al., 2001; Goldstein and Volkow, 2002; Li et al., 2012; Wang et al., 2014; Filbey et al., 2016; Allenby et al., 2020). Additionally, fMRI studies in humans have shown that (i) context/cue-induced anterior cingulate cortex (ACC) activation occurs selectively after exposure to drug-related context/cues, but not following exposure to natural rewards (Garavan et al., 2000), (ii) ACC activation is directly related to drug craving (Childress et al., 1999; Nora et al., 1999; Goldstein and Volkow, 2002; Bach et al., 2015), and (iii) individuals with greater ACC activation during drug-context/cue exposure are more likely to relapse (Allenby et al., 2020). Based on these findings, it has been posited, but not directly tested, that the ACC acts as a central hub that activates dopamine neurons within the ventral tegmental area (VTA) upon re-exposure to drug-associated contexts (Goldstein and Volkow, 2002). The result is an increase in craving and decreases in inhibitory control (Goldstein and Volkow, 2002).

To directly test the role of the ACC in context-induced drug seeking, we employed our novel behavioral procedure that is able to simultaneously measure the formation of context associations and reward-seeking behaviors (McKendrick et al., 2020a,b). Given that this procedure requires oral self-administration of drug, mice orally self-administered oxycodone, which is the typical route of administration for this opioid. Additional rationale for the importance of modeling oxycodone self-administration comes from clinical studies that have found that the majority of patients who misused opioids reported that their first opioid was a prescription drug (e.g., oxycodone) (Grau et al., 2007; Cicero et al., 2014). Moreover, oxycodone is highly addictive, likely through the agonistic effects on μ-opioid receptors and the ability to cross the blood-brain barrier *via* active transport, allowing for rapid increases in brain concentration and fast onset of action (Minhas and Leri, 2018). As a result, oxycodone misuse has become a major public health concern (O’Donnell et al., 2017). Based on this, we tested the hypothesis that the ACC and its connections to the VTA are important regulators of the formation of oxycodone-induced context associations and context-induced oxycodone seeking.

## Materials and methods

### Animals

All experiments were done in accordance with procedures approved by the Pennsylvania State University College of Medicine Institutional Animal Care and Use Committee. Mice used in this study included male C57BL/6 wild-type mice (aged 8–12 weeks). All mice were singly housed on a 12-h light/dark cycle with *ad libitum* food and water and were used for electrophysiology recordings (*n* = 71), behavioral experiments (*n* = 40), designer receptors exclusively activated by designer drug (DREADD) behavioral experiments (*n* = 70), or Cre-mediated DREADD behavioral experiments (*n* = 32). Animals were singly housed because in humans, it is known that social isolation increases vulnerability to substance use disorders (Newcomb and Bentler, 1988; Sinha, 2008), which often are accompanied by the development of drug-context associations (O’Brien and Ternes, 1986; O’Brien et al., 1992; Xue et al., 2012). Evidence suggests that socially isolated rodents are more vulnerable to developing drug-context associations (Whitaker et al., 2013), more reliably develop opioid context associations (Graziane et al., 2016; McDevitt and Graziane, 2019; McKendrick et al., 2020a,b), and are a better model of substance use disorders that are observed in humans.

### Drugs

Oxycodone hydrochloride was purchased from Sigma Aldrich (St. Louis, MO, United States) or was a gift from NIDA drug supply. Clozapine N-oxide (CNO) was a gift from NIDA drug supply.

### Stereotaxic surgery

Anesthesia was induced and maintained with isoflurane. The animal was placed in a stereotaxic frame (Stoelting, Wood Dale, IL, United States) and craniotomies were performed *via* microdrill. Injections were carried out *via* a 33-gauge beveled-tip needle [World Precision Instruments (WPI), Sarasota, FL, United States] connected to a 5 μl syringe (Hamilton Company, Reno, NV, United States) on a micro pump (Harvard Apparatus, Holliston, MA, United States) at an infusion rate of 100 nl/min for 3 min Following bilateral injection, the needle was left *in situ* for 5–10 min to allow for virus diffusion and then slowly retracted to limit backflow. For the first chemogenetic experiments, AAV5-hSyn-hM4D(Gi)-mCherry (Addgene #50475, titer: 8.6 × 10^12^ GC/ml) or AAV5-hSyn-mCherry (Addgene #11472; titer: 7 × 10^12^ vg/ml) was injected into the ACC [coordinates relative to Bregma (in mm): anterior-posterior (AP): +1.10; medial lateral (ML): ±0.325; dorsal ventral (DV): −2.00]. For inhibition of ACC projections to the VTA, mice were injected with a retrograde-Cre recombinase (pENN.AAV.hSyn.Cre.WPRE.hGH; Addgene #105553-AAVrg; titer: 2.39 × 10^13^ GC/ml) in the VTA [coordinates relative to Bregma, anterior-posterior (AP) −3.00 mm; medial lateral (ML) ± 0.50 mm; dorsal ventral (DV) −4.50 mm]. Four weeks later, mice were either injected with a Cre-dependent vehicle control (AAV5-hSyn-DIO-mCherry; Addgene #50459; titer: 1.1 × 10^13^ GC/ml) or a Cre-dependent hM4di virus in experimental mice [AAV5-hSyn-DIO-hM4di(Gi)-mCherry; Addgene #44362; titer: 1.1 × 10^13^ GC/ml]. Mice were used for behavioral experiments 4 weeks post-injection to allow for virus expression. All viral vectors were a gift from Bryan Roth.

### Oxycodone oral self-administration procedure

Oxycodone oral self-administration was performed based on our previously published procedure (McKendrick et al., 2020a,b) using a mouse 3-compartment chamber (Med Associates, St. Albans, VT, United States).

#### Habituation

Mice were placed in the center compartment with free access to all three compartments for 20 min once a day for 2 days. Time spent (seconds) in each compartment and total activity counts were recorded and averaged. The average time spent in each compartment following the two habituation sessions was used to determine baseline preference.

#### Conditioning

Drinking bottles were created as described (McKendrick et al., 2020b). Briefly, we modified 10 ml serological pipettes by tapering both ends, placing a stainless-steel sipper tube (Ancare; OT-300) in one end and a silicone stopper (Thermo Fisher Scientific; 09-704-1D) in the other. Bottles were inserted into plastic holders that were then placed directly into CPP chambers, where they were positioned so that the sipper was ∼5 cm above the chamber floor. Pennsylvania State University Fabrication shop constructed plexiglass tops that were placed along the top of the 3-compartment apparatus and allowed for plastic bottle holders to be placed into chambers. Oral self-administration was recorded as the ml before and following all sessions. We utilized a biased approach in which 0.2% (w/v) saccharin solution (control group) or a low dose (0.1 mg/ml), middle dose (0.5 mg/ml), or high dose (1.0 mg/ml) of oxycodone diluted in 0.2% saccharin solution (experimental group) was placed in the least-preferred context. 0.2% saccharin solution was used as a vehicle to overcome the bitter taste that accompanies prototypical opioids making up the opioid chemical class phenanthrene. Additionally, we and others have shown that 0.2% saccharin solution does not evoke CPP (White and Carr, 1985; Stefurak and van der Kooy, 1992; Agmo and Marroquin, 1997). Twenty four to seventy-two hours after habituation, mice underwent 6 overnight (ON) sessions (14 h sessions) of conditioning (ON1–ON6) with odd nights (ON1, ON3, and ON5) corresponding to access to water (control group) or 0.2% saccharin (experimental group) in the most preferred side of the CPP chamber. Even nights (ON2, ON4, and ON6) corresponded to access to 0.2% saccharin (control group) or the low (0.1 mg/ml), middle (0.5 mg/ml), or high (1.0 mg/ml) dose oxycodone (experimental group) in the least preferred chamber. Each morning, mice were placed back into their home cage.

#### Post-conditioning (extinction test)

Twenty-four or forty-eight hours after the end of the last conditioning session, mice underwent CPP and extinction testing. Here, mice were placed in the 3-compartment chamber and allowed to move freely for 1 h (tests in involving DREADD activation with CNO) or 2 h (tests in mice not expressing DREADDs). Bottles were filled with water and present in both chambers. CPP scores were calculated as time spent on the least preferred side on test day minus the average time spent on the same side during preconditioning during the first 20 min of the test. Milliliters of water consumed in both chambers was measured after completion of the 1 or 2 h test.

### Locomotor activity

Locomotor activity was tracked during conditioning sessions *via* automated software (Med Associates, St. Albans VT, United States) controlling infrared light sensors within the CPP apparatus (Med Associates, St. Albans VT, United States). Locomotor activity was recorded throughout the entire duration of the overnight session as well as during the entirety of the extinction test.

### Spontaneous opioid withdrawal

Spontaneous opioid withdrawal was assessed using previously described methods (Papaleo and Contarino, 2006; Madayag et al., 2019; McDevitt et al., 2021). Briefly, 24–28 h after the final conditioning session, a time range used throughout this study, which encompasses the expression of significant spontaneous opioid withdrawal (Papaleo and Contarino, 2006; Madayag et al., 2019), mice were placed into a clear acrylic open field arena (37 cm × 23 cm × 15 cm) and examined for signs of somatic withdrawal during a 30-min period. A 30 min period was chosen because this duration is commonly used to asses spontaneous opioid withdrawal in rodents (Papaleo and Contarino, 2006). This time point remains consistent with the 30 min duration employed for naloxone-precipitated withdrawal, which is based on naloxone’s short half-life (Carmack et al., 2019). Experimenters were blinded to treatment. A total withdrawal score was calculated as follows: # of jumps + # of tremors + # of grooming sessions + # of paw flutters + piloerection (scored as either 1 or 0, presence or absence). These phenotypes were included in the withdrawal score as they have been shown to be correlated to withdrawal-like symptoms in rodents (Papaleo and Contarino, 2006; Madayag et al., 2019).

### Anterior cingulate cortex chemogenetic inhibition

To assess the role of ACC activity during early opioid withdrawal, we inhibited the ACC with the G_*i/o*_-coupled hM4Di DREADD, and in separate experiments, the ACC projections to the VTA using a Cre-dependent G_*i/o*_-coupled hM4di DREADD. Clozapine N-oxide (CNO) was dissolved in saline with 2% DMSO. For hM4di-treated mice and the corresponding control group, a total of 70 mice were used. On day 1 of abstinence, vehicle controls and G_*i/o*_-coupled hM4Di DREADD-expressing mice were i.p. injected with 2 mg/kg CNO 15 min prior to the start of withdrawal monitoring (*n* = 40) or a CPP and a 1 h extinction test (*n* = 30). This time point is suitable for adequate CNO penetration in the mouse brain (Jendryka et al., 2019). For experiments involving Cre-dependent G_*i/o*_-coupled hM4di DREADD, 24 h following the last conditioning session, mice were i.p. injected with 2 mg/kg CNO 15 min prior to the start of withdrawal monitoring. One day later (i.e., 48 h following the last conditioning session), mice were re-injected with 2 mg/kg CNO to re-establish brain CNO levels and 15 min later were assessed for CPP (20 min) and drug seeking (1 h). Based on DREADD activation following CNO injection (∼70 min) (Chang et al., 2015), 1 h extinction sessions were performed.

### Acute brain slice preparation

Following the extinction test (the post-conditioning test when no drug was present), mice were deeply anesthetized with isoflurane and cardiac perfused with an ice-cold NMDG-based cutting solution containing (in mM): 135 N-methyl-D-glucamine, 1 KCl, 1.2 KH_2_PO_4_, 0.5 CaCl_2_, 1.5 MgCl_2_, 20 choline-HCO_3_, and 11 glucose, saturated with 95% O_2_/5% CO_2_, adjusted to a pH of 7.4 with HCl, osmolality adjusted to 305 mmol/kg. Following perfusion, mice were decapitated and brains were rapidly removed. 250 μm coronal brain slices containing the ACC were prepared *via* a Leica VT1200s vibratome in 4°C NMDG cutting solution. Following cutting, slices were allowed to recover in artificial cerebrospinal fluid (aCSF) containing (in mM): 119 NaCl, 2.5 KCl, 2.5 CaCl2, 1.3 MgCl_2_, 1 NaH_2_PO_4_, 26.2 NaHCO_3_, and 11 glucose, osmolality of 290 mmol/kg, at 31°C for 30 min followed by 30 min at 20–22°C prior to recording. After a 1 h recovery period, slices were kept at 20–22°C for the rest of the recording day.

### Electrophysiology

All whole-cell electrophysiology recordings were made from Layer 5 (L5) ACC pyramidal neurons (PyNs) spanning between Bregma +1.10 and +0.62. PyNs were identified *via* infrared differential interference contrast microscopy and their cell properties. In this study, we first focused on L5 PyNs that had low membrane resistance (<100 MΩ), high capacitance (>150 pF), and predominate voltage sag, as these have been shown to project subcortically to the thalamus, tectum, brainstem, and spinal cord. We found this subtype of L5 PyN to be best described morphologically as “thick-tufted,” with HCN channel-mediated current, large somas, and likely expressing dopamine D2 receptors (Anastasiades and Carter, 2021; McDevitt et al., 2021). We also examined VTA-projecting ACC neurons as well as L5 “thin-tufted” PyNs which had higher membrane resistance (>150 MΩ), lower capacitance (<100 pF), and no or a small voltage sag, which are putative dopamine D1 receptor expressing (Gee et al., 2012).

For measurements of intrinsic membrane excitability (IME), rheobase, and intrinsic properties, recording electrodes [3–5 MΩ; borosilicate glass capillaries (WPI #1B150F-4)] were pulled on a horizontal puller from Sutter Instruments (model P-97) and filled with a potassium-based internal solution containing (in mM): 130 KMeSO_3_, 10 KCl, 10 HEPES, 0.4 EGTA, 2 MgCl_2_–6H_2_0, 3 Mg-ATP, 0.5 Na-GTP, pH 7.2–7.4, osmolality = 290 mmol/kg (Wescor Vapro Model 5,600, ElitechGroup).

Resting membrane potential was recorded immediately following break-in. For IME experiments, we employed a commonly used current step protocol (Ishikawa et al., 2009; McDevitt and Graziane, 2019; McDevitt et al., 2019; Roselli et al., 2020). The IME and rheobase protocols were conducted at unadjusted resting membrane potentials as previously performed in the ACC (McDevitt et al., 2021). Our current step protocol, consisting of 1 s steps ranging from −150 to +400 pA in 50 pA increments, was carried out with a 15 s intra-sweep interval. The number of action potentials observed at each current step was recorded. For experiments where CNO (3 μM) was bath applied, IME was measured first in the absence and then in presence of CNO, as performed previously (McDevitt et al., 2021). For rheobase experiments, a 2 s consistent-slope current injection ramp with a maximal current of 400 pA was performed, as previously described (Pati et al., 2020). The rheobase was defined as the minimal current needed to elicit an action potential.

All recordings were performed in 30–32°C aCSF using either an Axon Multiclamp 700B amplifier or Sutter Double IPA, filtered at 2–3 kHz, and digitized at 20 kHz. Series resistance was typically 10–25 MΩ, left uncompensated, and monitored throughout. For all current-clamp recordings, cells with a bridge balance that varied greater than 20% during the start and end of recordings were discarded from analysis.

### Statistical analysis

All results are shown as mean ± SEM. Each experiment was replicated in at least three animals. No data points were excluded. The sample size was presented as n/m, where “n” refers to the number of cells and “m” refers to the number of animals. Statistical significance was assessed in GraphPad Prism software (9.1.2) using one- or two-way ANOVAs with Bonferroni’s correction for multiple comparisons in order to identify differences as specified. *F*-values for two-way ANOVA statistical comparisons represent interactions between variables unless otherwise stated. Two-tail tests were performed for all studies.

## Results

### Oxycodone oral self-administration evokes conditioned place preference and context-induced drug-seeking

**FIGURE 1:**
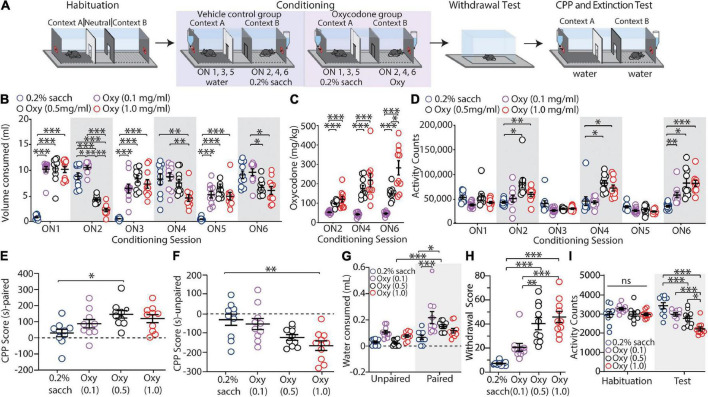
Oxycodone oral self-administration evokes conditioned place preference, context-induced drug-seeking, and withdrawal. **(A)** Timeline of the behavioral procedure of saccharin control or oxycodone oral-SA-CPP. Mice underwent habituation sessions followed by 6 overnight conditioning sessions where they were given access to water (control) or saccharin (experimental) in the unpaired chambers and saccharin (control) or oxycodone diluted in saccharin solution (experimental) in the paired chambers. 1-2 days later, mice were given withdrawal testing followed by a 2 h extinction test. **(B)** Summary graph showing the volume of solution consumed between groups during overnight (ON) conditioning sessions 1-6 (ON 1-6) [*F*_(15,180)_ = 29.84, *p* < 0.0001, two-way repeated-measures ANOVA, Bonferroni *post hoc* test]. **(C)** Summary graph showing the mg/kg of oxycodone consumed during ON 2, 4, and 6 [*F*_(4,54)_ = 13.49, *p* < 0.001, two-way repeated-measures ANOVA, Bonferroni *post hoc* test]. (**D**) Summary graph showing the locomotor activity counts across conditioning sessions [*F*_(15,180)_ = 7.605, *p* < 0.0001, two-way repeated-measures ANOVA, Bonferroni *post hoc* test]. **(E)** Summary graph showing the CPP score for all groups in the paired chamber [*F*_(3,36)_ = 4.215, *p* = 0.0118, one-way ANOVA with Bonferroni *post hoc* test]. **(F)** Summary graph showing the CPP score for all groups in the unpaired chamber [*F*_(3,36)_ = 6.428, *p* = 0.0016, one-way ANOVA with Bonferroni *post hoc* test]. **(G)** Summary graph showing mLs of water consumed during the 2 h extinction test [*F*_(3,72)_ = 2.743, *p* = 0.0493, two-way ANOVA with Bonferroni *post hoc* test]. **(H)** Summary graph showing the withdrawal scores for all groups [*F*_(3,36)_ = 21.88, *p* < 0.0001, one-way ANOVA with Bonferroni *post hoc* test]. **(I)** Summary graph showing total activity counts during habituation and 24 h following the last conditioning session [*F*_(3,36)_ = 24.29, *p* < 0.0001, two-way repeated-measures ANOVA with Bonferroni *post hoc* test] (n = 10 for each group). *p < 0.05, ^**^p < 0.001, ^***^p < 0.0001.

In order to investigate the role of the ACC in context-induced oxycodone seeking, we employed a preclinical model that combines self-administration with measurable outcomes related to the formation of context associations, which we have previously shown is effective at simultaneously measuring drug-seeking and CPP (McKendrick et al., 2020a,b). Here, mice were given six overnight sessions, in which controls (0.2% sacch) received access to water in the most-preferred chamber (unpaired chamber) and a 0.2% saccharin solution (vehicle control) in the least-preferred chamber (paired chamber). Experimental mice (Oxy) were given access to the vehicle control 0.2% saccharin solution in the most-preferred chamber (unpaired chamber) and a low dose (0.1 mg/ml), middle dose (0.5 mg/ml) or high dose (1.0 mg/ml) oxycodone solution dissolved in 0.2 % saccharin in the least-preferred compartment (paired chamber) (Figure 1A).

First, we monitored the volume of solution consumed during each overnight (ON) session. We observed that middle (0.5 mg/ml) and high (1.0 mg/ml) dose oxycodone self-administering mice increased their consumption of oxycodone during each overnight session in the drug-paired chamber (ON 2, 4, and 6) (0.5 mg/ml: ON2 vs. ON6: *p* = 0.0018; 1.0 mg/ml: ON2 vs. ON6: *p* = 0.0054; two-way repeated measures ANOVA with Bonferroni *post hoc* test), while decreasing their consumption of saccharin solution when placed in the unpaired chamber (ON 1, 3, and 5) (0.5 mg/ml: ON1 vs. ON5: *p* = 0.0217; 1.0 mg/ml: ON1 vs. ON5: *p* = 0.0021; two-way repeated measures ANOVA with Bonferroni *post hoc* test) (Figure 1B). Corresponding to this, we observed an escalation of oxycodone consumption measured in mg/kg among the middle (0.5 mg/ml) and high (1.0 mg/ml) dose oxycodone groups (0.5 mg/ml: ON2 vs. ON6: *p* = 0.0004; 1.0 mg/ml: ON2 vs. ON6: *p* = 0.0009; two-way repeated measures ANOVA with Bonferroni *post hoc* test) (Figure 1C). We did not observe any difference in 0.2% saccharin consumption in control animals during each overnight session when 0.2% sacch was available (ON 2, 4, and 6) (ON2 vs. ON6: *p* > 0.9999; two-way repeated measures ANOVA with Bonferroni *post hoc* test) (Figure 1B). Overall, these results suggest that mice who self-administered the middle (0.5 mg/ml) and high (1.0 mg/ml) dose of oxycodone developed tolerance to opioids as well as a shift in preference from 0.2% sacch solution to oxycodone-containing solution during the training sessions.

Given that oxycodone exposure increases activity in rodents (Niikura et al., 2013), we verified that mice were self-administering oxycodone by monitoring locomotor activity counts during overnight sessions 2, 4, and 6. Our results show that by ON 6, all oxycodone groups (0.1, 0.5, and 1.0 mg/ml) had significant increases in activity counts compared to saccharin controls (0.1 mg/ml: *p* = 0.0048; 0.5 mg/ml: *p* = 0.0030; 1.0 mg/ml: *p* < 0.0001; two-way repeated measures ANOVA with Bonferroni *post hoc* test) (Figure 1D).

Twenty-four or forty-eight hours following the last overnight conditioning session, mice underwent a CPP test and 2 h extinction test with water given in both the paired and unpaired chambers. This allowed us to test the time spent in the drug-paired and unpaired-context (the formation of oxycodone-induced context associations) during the first 20 min of the test as well as the volume of water consumed in each compartment (oxycodone-seeking behavior) following the 2 h extinction test.

In monitoring CPP, our results show that there was a significant increase in the amount of time spent in the oxycodone-paired chamber in mice who self-administered the middle dose (0.5 mg/ml) of oxycodone compared to saccharin drinking control mice (0.5 mg/ml: *p* = 0.0123; one-way ANOVA with Bonferroni *post hoc* test) (Figure 1E). We did not observe a significant difference in the CPP score in the low (0.1 mg/ml) or high (1.0 mg/ml) dose oxycodone mice (0.1 mg/ml: *p* = 0.4644; 1.0 mg/ml: *p* = 0.0578; one-way ANOVA with Bonferroni *post hoc* test) (Figure 1E). We also observed that there was a significant decrease in the amount of time spent in the unpaired chamber in mice who self-administered the high dose (1.0 mg/ml) of oxycodone compared to saccharin controls (*p* = 0.0027; one-way ANOVA with Bonferroni *post hoc* test) (Figure 1F). In contrast, we found no significant difference in the time spent in the unpaired chamber for mice that self-administered 0.1 mg/ml or 0.5 mg/ml of oxycodone compared to control mice (0.1 mg/ml: *p* > 0.9999; 0.5 mg/ml: *p* = 0.0795; one-way ANOVA with Bonferroni *post hoc* test) (Figure 1F).

In monitoring drug-seeking behavior as measured by the volume of water consumed from each chamber during the 2 h extinction test, we observed a significant increase in volume of water consumed in oxycodone-paired chambers in both the low (0.1 mg/ml) and middle (0.5 mg/ml) dose oxycodone groups compared to the volume of water consumed in the paired-context of the control group (0.1 mg/ml: *p* < 0.0001; 0.5 mg/ml: *p* = 0.0245; two-way ANOVA with Bonferroni *post hoc* test) (Figure 1G). Additionally, we observed that the middle dose (0.5 mg/ml) oxycodone group consumed significantly more water in the oxycodone-paired chamber versus the unpaired chamber (*p* < 0.0001; two-way ANOVA with Bonferroni *post hoc* test) (Figure 1G). We did not observe a significant difference in water consumed in the paired chamber when comparing the high dose (1.0 mg/ml) oxycodone group versus the control group (*p* = 0.4858; two-way ANOVA with Bonferroni *post hoc* test) (Figure 1G). Overall, these results demonstrate that mice that orally self-administered oxycodone at 0.5 mg/ml express CPP and drug seeking in the oxycodone paired context.

### Oxycodone oral self-administration facilitates spontaneous opioid withdrawal

To investigate whether mice self-administering oxycodone became opioid dependent, we performed withdrawal tests during the one-day abstinence time point. We found that the middle (0.5 mg/ml) and high (1.0 mg/ml) dose oxycodone groups had significantly higher withdrawal scores compared to the control group who self-administered 0.2% saccharin in the paired chamber (0.5 mg/ml: *p* < 0.0001; 1.0 mg/ml: *p* < 0.0001; one-way ANOVA Bonferroni *post hoc* test) (Figure 1H). In contrast, we found no significant difference in spontaneous opioid withdrawal when comparing the low dose (0.1 mg/ml) oxycodone group versus controls (*p* = 0.1085; one-way ANOVA Bonferroni *post hoc* test) (Figure 1H).

We next ran an additional test to monitor locomotor activity during withdrawal as it has been previously shown that mice undergoing spontaneous opioid withdrawal have decreased locomotor activity (Madayag et al., 2019; McDevitt et al., 2021). For these experiments, we compared the total activity counts during the extinction test, which occurred 24 h following the last conditioning session. We found that on test day, middle (0.5 mg/ml) and high (1.0 mg/ml) dose oxycodone-treated mice had significant decreases in activity counts compared to control mice (0.5 mg/ml: *p* = 0.0009; 1.0 mg/ml: *p* < 0.0001; two-way repeated measures ANOVA with Bonferroni *post hoc* test) (Figure 1I). Additionally, the high dose (1.0 mg/ml) oxycodone group had significantly fewer activity counts than both low (0.1 mg/ml) and middle (0.5 mg/ml) dose oxycodone mice (vs. 0.1 mg/ml: *p* < 0.0001; vs. 0.5 mg/ml: *p* = 0.0123) (Figure 1I). In line with this, we found that during conditioning, when oxycodone mice were exposed to 0.2% saccharin in the unpaired chamber, only the high dose (1.0 mg/ml) oxycodone group expressed a significant decrease in activity when comparing ON 3 vs. ON 5 (0.1 mg/ml: *p* > 0.9999; 0.5 mg/ml: *p* > 0.9999; 1.0 mg/ml: *p* = 0.0306; two-way repeated measures ANOVA with Bonferroni *post hoc* test).

Overall, using this oral-SA CPP method, we were able to evoke oxycodone-induced place preference, context-induced drug seeking, and spontaneous opioid withdrawal in oxycodone-treated mice using the middle dose of oxycodone (0.5 mg/ml). As such, we used 0.5 mg/ml of oxycodone for the remaining experiments.

### Anterior cingulate cortex regulates oxycodone-induced conditioned place preference, and context-induced drug seeking, and opioid-induced spontaneous withdrawal

**FIGURE 2:**
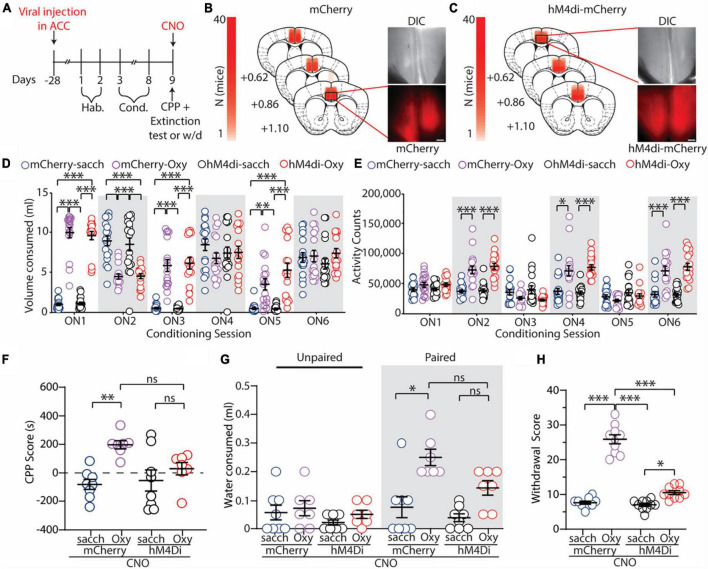
Global inhibition of the ACC attenuates oxycodone-induced CPP, context-induced drug seeking, and spontaneous opioid withdrawal. **(A)** Timeline of the experimental paradigm. **(B,C)** Summary demonstrating viral injection placements in mCherry **(B)** and hM4di-expressing **(C)** mice. Scale bars = 200 μm. **(D)** Summary graph showing the volume of solution consumed between groups during overnight conditioning sessions (ON1-6) [*F*_(15,330)_ = 29.47; *p* < 0.0001; two-way repeated measures ANOVA with Bonferroni *post hoc* test] (mCherry-sacch: *n* = 18; mCherry-Oxy: *n* = 17; hM4di-sacch: *n* = 18; hM4di-Oxy: *n* = 17). **(E)** Summary graph showing the locomotor activity counts across conditioning sessions [*F*_(15,330)_ = 15.24; *p* < 0.0001; two-way repeated measures ANOVA with Bonferroni *post hoc* test] (mCherry-sacch: n = 18; mCherry-Oxy: n = 17; hM4di-sacch: n = 18; hM4di-Oxy: *n* = 17). **(F)** Summary graph showing the CPP score in control and oxycodone-conditioned mice with mCherry or hM4di expression in the ACC. Experiments were performed in the presence of CNO (2 mg/kg, i.p.) [*F*_(3,26)_ = 6.095; *p* = 0.0028; one-way ANOVA with Bonferroni *post hoc* test] (mCherry-sacch: *n* = 8; mCherry-Oxy: *n* = 7; hM4di-sacch: *n* = 8; hM4di-Oxy: *n* = 7). **(G)** Summary graph showing the milliliters of water consumed in the unpaired and paired chambers by control and oxycodone-conditioned mice with mCherry or hM4di expression in the ACC. Experiments were performed in the presence of CNO (2 mg/kg, i.p.) [*F*_(3,52)_ = 4.944; *p* = 0.0043; two-way ANOVA with Bonferroni *post hoc* test] (mCherry-sacch: *n* = 8; mCherry-Oxy: *n* = 7; hM4di-sacch: *n* = 8; hM4di-Oxy: *n* = 7). **(H)** Summary graph showing withdrawal score of control and oxycodone-conditioned mice with mCherry or hM4di expression in the ACC. Experiments were performed in the presence of CNO (2 mg/kg, i.p.) [*F*_(3,36)_ = 132.7; *p* < 0.0001; one-way ANOVA with Bonferroni *post hoc* test] (mCherry-sacch: *n* = 10; mCherry-Oxy: *n* = 10; hM4di-sacch: *n* = 10; hM4di-Oxy: *n* = 10]. *p < 0.05, ^**^p < 0.001, ^***^p < 0.0001.

Next, employing our oral-SA-CPP paradigm, we aimed to directly test whether the ACC is causally associated with opioid-seeking behaviors. To test this, we globally inhibited ACC function using chemogenetic approaches as we have previously shown that this approach is effective at inhibiting hM4di-expressing ACC neurons in the presence of CNO (McDevitt et al., 2021). Four weeks prior to the onset of behavioral experiments, mice (*n* = 70) were injected bilaterally in the ACC with a viral construct containing either an mCherry fluorophore (vehicle controls; mCherry) or an inhibitory hM4di(Gi) DREADD construct conjugated to the mCherry fluorophore (hM4di) (Figure 2A). Expression of these viral constructs was confirmed at the conclusion of the experiments (Figures 2B,C). For these experiments, mice expressing mCherry or hM4di viral constructs underwent oral-SA-CPP training. Control groups received 0.2% saccharin (sacch) in the paired chamber (water in the unpaired chamber) and the experimental groups received oxycodone (0.5 mg/ml) diluted in 0.2% saccharin solution (Oxy) in the paired chamber (0.2% saccharin in the unpaired chamber) (Figure 2A). Mice then underwent CPP and 1 h extinction test in the presence of CNO (2 mg/kg) or withdrawal testing in the presence of CNO (2 mg/kg) 24 h following the last conditioning session.

Similar to what we observed previously during oral-SA-CPP conditioning (Figure 1), we found that mice who self-administered oxycodone (0.5 mg/ml) increased their consumption of oxycodone during each overnight session in the paired chamber (ON 2, 4, and 6) (mCherry-Oxy: ON2 vs. ON6: *p* = 0.047; hM4di-Oxy: ON2 vs. ON6: *p* = 0.007; two-way repeated measures ANOVA with Bonferroni *post hoc* test) (Figure 2D), while decreasing their consumption of saccharin solution when placed in the unpaired chamber (ON 1, 3, and 5) (mCherry-Oxy: ON1 vs. ON5: *p* < 0.0001; hM4di-Oxy: ON1 vs. ON5: *p* = 0.0017; two-way repeated measures ANOVA with Bonferroni *post hoc* test) (Figure 2D). During conditioning, we did not observe a significant difference in consumption of 0.2% saccharin in the control groups on ON 2 versus 6 (mCherry-sacch: ON2 vs. ON6: *p* = 0.085; hM4di-sacch: ON2 vs. ON6: *p* = 0.3926; two-way repeated measures ANOVA with Bonferroni *post hoc* test) (Figure 2D). Additionally, we again observed that mice who orally self-administered oxycodone (0.5 mg/ml) on ON 2, 4, and 6 displayed significant increases in locomotor activity compared to controls (*F*_(15,330)_ = 15.24, *p* < 0.0001; two-way repeated measures ANOVA with Bonferroni *post hoc* test) (Figure 2E).

Following conditioning, we tested the effects of global ACC inhibition on the expression of drug-context associations and on context-induced drug seeking. 24 h following the last conditioning session, mice were injected with CNO (2 mg/kg) 15 min prior to the CPP and 1 h extinction test. CNO was injected to evoke activation of the inhibitory hM4di DREADD in the ACC and a 1 h extinction test was used to correspond to the time course of DREADD activation prior to CNO delivery (∼70 min) (Chang et al., 2015). Our results show that mCherry-Oxy mice expressed a significant increase in the CPP score compared to mCherry-sacch controls (*p* = 0.0035; one-way ANOVA with Bonferroni *post hoc* test) (Figure 2F). However, we found that inhibition of the ACC attenuated oxycodone-induced place preference because there was no significant difference in CPP scores between hM4di-Oxy and hM4di-sacch mice (*p* > 0.999; one-way ANOVA with Bonferroni *post hoc* test) as well as hM4di-Oxy and mCherry-Oxy (*p* = 0.184; one-way ANOVA with Bonferroni *post hoc* test) (Figure 2F).

To investigate drug-seeking behaviors, we measured the volume of water consumed from the unpaired and drug-paired chambers during the 1 h extinction test. We found that mCherry-Oxy mice consumed significantly more water in the drug-paired chamber compared to the mCherry-sacch controls (*p* = 0.0001; one-way ANOVA with Bonferroni *post hoc* test) (Figure 2G). Additionally, we found that ACC inhibition attenuated context-induced drug-seeking behavior as there was no significant difference in water consumed between hM4di-Oxy and hM4di-sacch mice (*p* = 0.0972; two-way ANOVA with Bonferroni *post hoc* test) as well as hM4di-Oxy and mCherry-Oxy (*p* = 0.1055; two-way ANOVA with Bonferroni *post hoc* test) (Figure 2G). Overall, we observed that inhibiting global ACC activity served to attenuate both oxycodone-induced CPP and drug-seeking behaviors.

Lastly, given that we have previously shown that global inhibition of the ACC attenuates morphine-induced spontaneous withdrawal (McDevitt et al., 2021), we measured the effects of ACC inhibition on spontaneous opioid withdrawal evoked by oral self-administration of oxycodone (0.5 mg/ml). 24 h following the last conditioning session, mice were treated with CNO (2 mg/kg) 15 min prior to withdrawal testing. Here, we observed that the mCherry-injected mice conditioned with oxycodone had a significantly higher withdrawal score than both mCherry-sacch and hM4di-sacch controls (*p* < 0.0001 and *p* < 0.0001, respectively; one-way ANOVA with Bonferroni *post hoc* test) (Figure 2H). Furthermore, we found that globally inhibiting the ACC significantly decreased withdrawal scores in the hM4di-Oxy mice compared to the mCherry-Oxy mice (*p* < 0.0001; one-way ANOVA with Bonferroni *post hoc* test) (Figure 2H). However, as we have observed previously (McDevitt et al., 2021), ACC inhibition did not entirely block the withdrawal phenotype, as the hM4di-Oxy mice still had a significantly higher withdrawal score compared to hM4di-sacch controls (*p* = 0.0162; one-way ANOVA with Bonferroni *post hoc* test) (Figure 2H).

### Anterior cingulate cortex L5 thick-tufted, but not thin-tufted, PyN intrinsic excitability is enhanced during 1 day abstinence from oxycodone self-administration

**FIGURE 3:**
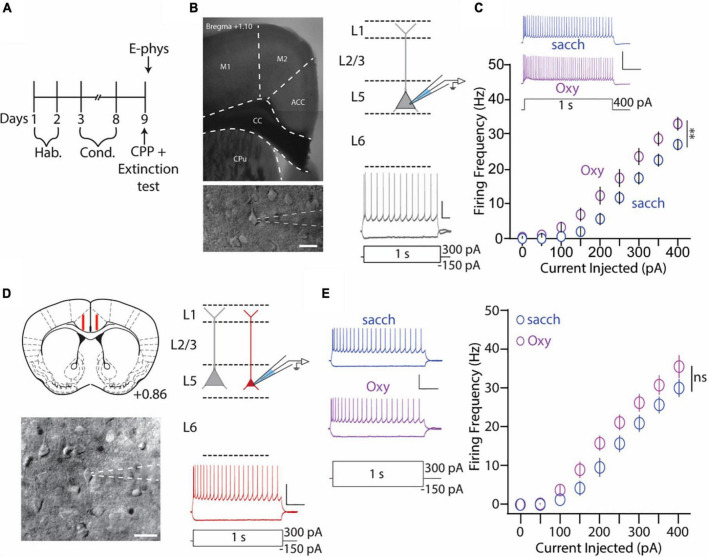
Anterior cingulate cortex (ACC) L5 thick-tufted, but not thin-tufted, PyNs have increase in the intrinsic membrane excitability during 1 day abstinence from oxycodone (0.5 mg/ml) self-administration. **(A)** Experimental timeline showing that electrophysiology assessments were performed immediately after the 2 h extinction test. **(B)** 4× image of a brain slice containing the ACC (top) and 40× image of an ACC thick-tufted pyramidal neuron targeted for electrophysiological assessments (bottom). These neurons are located within layer 5 of the ACC (top, right) and express a SAG current (bottom, right). Scale bar (image): 20 μm. Scale bar (electrophysiology trace): 25 mV, 100 ms. **(C)** Summary graph showing that following the 2 h extinction test, the number of action potentials fired in L5 thick-tufted PyNs is significantly increased in mice orally self-administering oxycodone vs. vehicle controls. Scale bars: 50 mV, 200 ms [*F*_(8,208)_ = 2.615, *p* = 0.0095, two-way repeated-measures ANOVA with Bonferroni *post hoc* test] (saccharin: *n* = 12/4; oxycodone: *n* = 16/4; cells/mice). **(D)** Illustration of the targeted region in red (layer 5) for recording thin-tufted pyramidal neurons in a brain slice containing the ACC. (Bottom) Illustration of a thin-tufted PyN targeted for electrophysiology recordings with distinct morphological (top, right) and electrophysiological (bottom, right) electrophysiological characteristics. Scale bar (image): 20 μm. Scale bar (electrophysiology trace): 50 mV, 200 ms. **(E)** Representative traces (left) and summary graph (right) showing no significant difference in IME following oxycodone SA in ACC L5 thin-tufted PyNs [*F*_(8,120)_ = 1.689, *p* = 0.1078, two-way repeated-measures ANOVA with Bonferroni *post hoc* test] (saccharin: *n* = 8/3; oxycodone: *n* = 9/3; cells/mice). 50 mV, 200 ms. ^**^p < 0.01.

We have shown that global inhibition of the ACC attenuates context-induced drug-seeking behaviors in a preclinical model (Figure 2). Based on these findings, we next wanted to investigate whether specific types of neurons are activated within the ACC upon re-exposure to drug-paired contexts. For these experiments, mice underwent the same oxycodone oral-SA-CPP method as described in Figure 1. Following extinction testing, when mice were re-exposed to the drug-paired context, mice were euthanized and brain slices containing the ACC were prepared for electrophysiological assessments (Figure 3A). We focused our assessment on thick- and thin-tufted of pyramidal neurons in Layer 5 (L5) in the ACC, each with distinct intrinsic properties (Table 1). We focused on these two types of pyramidal neurons based on their projections to brain regions involved in motivation, reward, and withdrawal (Gee et al., 2012; Seong and Carter, 2012; Dembrow and Johnston, 2014; Elston and Bilkey, 2017; Radnikow and Feldmeyer, 2018; van Heukelum et al., 2020). To assess neuronal excitability, we measured the number of action potentials generated during depolarizing current injections, first on ACC L5 thick-tufted PyNs, identified through electrophysiological and morphological characteristics (Figure 3B). We observed significantly more action potentials generated in mice who underwent oxycodone conditioning compared to saccharin controls (*F*_(8,208)_ = 2.615, *p* = 0.0095, two-way repeated-measures ANOVA with Bonferroni *post hoc* test) (Figure 3C), which was in contrast to thin-tufted pyramidal neurons, which showed no changes in the intrinsic membrane excitability between oxycodone and control groups (*F*_(8,120)_ = 1.689, *p* = 0.1078, two-way repeated-measures ANOVA with Bonferroni *post hoc* test) (Figures 3D,E). Overall, these results suggest that re-exposure to drug-associated contexts during 1 d abstinence from oxycodone self-administration evoke activation in the ACC in a specific class of L5 pyramidal neurons.

**TABLE 1:** Electrophysiological properties of thick- and thin-tufted PyNs.

Properties	Thick-tufted PyNs		*p* value	Thin-tufted PyNs		*p* value
	0.2% saccharin (*n* = 12)	Oxycodone (0.5 mg/ml) (*n* = 16)		0.2% saccharin (*n* = 8)	Oxycodone (0.5 mg/ml) (*n* = 10)	
RMP	−73.39 ± 0.94	−71.89 ± 0.80	0.2316	−79.41 ± 1.15	−76.97 ± 1.30	0.1891
Capacitance	160.8 ± 6.2	141.6 ± 7.7	0.0780	104.9 ± 9.3	80.36 ± 5.9	0.0334*
Resistance	110.7 ± 7.1	127.2 ± 8.6	0.1716	181.5 ± 19.8	288.1 ± 18.4	0.0012**
Rheobase	170.9 ± 15.2	145.3 ± 15.6	0.2521	167.4 ± 12.8	107.2 ± 9.14	0.0012**

p Value calculated using a Student’s unpaired t-test.

*p < 0.05,**p < 0.01.

### Anterior cingulate cortex neuronal projections to the ventral tegmental area are necessary for the expression of oxycodone withdrawal, place preference, and drug-seeking behaviors

We next aimed to directly test whether VTA-projecting ACC neurons possess increased excitability following re-exposure to drug-paired contexts and whether these neurons regulate context-induced drug-seeking behaviors.

**FIGURE 4:**
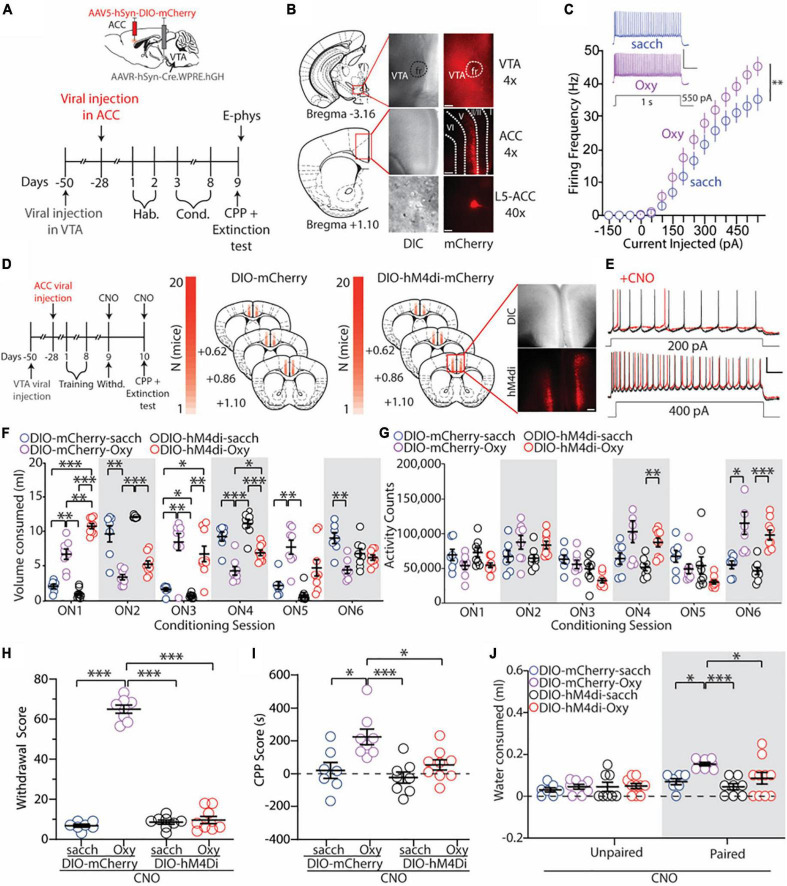
Inhibition of ACC neurons that project to the VTA blocks the expression of oxycodone withdrawal, place preference, and drug-seeking behaviors. **(A)** Experimental timeline showing that electrophysiology assessments were performed immediately after the 1 h extinction test in control or oxycodone (0.5 mg/ml) self-administering mice injected with DIO-mCherry bilaterally in the ACC. **(B)** 4× images of viral expression in the VTA (top) and ACC (middle). (Bottom) 40× image of a fluorescently-labeled VTA-projecting neuron in layer 5 of the ACC. Dashed lines indicate cortical layers I, II/III, V, and VI. Scale bars = 200 μm (4×) and 20 μm (40×). **(C)** Summary graph showing that following the 1 h extinction test, the number of action potentials fired in VTA-projecting ACC neurons is significantly increased in mice who orally self-administer oxycodone vs. vehicle controls. Scale bars: 50 mV, 200 ms [*F*_(14,336)_ = 2.551, *p* = 0.0017, two-way repeated-measures ANOVA with Bonferroni *post hoc* test] (DIO-mCherry-sacch: *n* = 12/4; DIO-mCherry-Oxy: *n* = 14/4; cells/mice). Scale bars = 50 mV, 0.2 s. **(D)** Experimental timeline and placements of viral injections in mice injected with DIO-mCherry or DIO-hM4di. Scale bars = 200 μm. **(E)** Representative traces showing bath application of CNO (3 μM) decreases the excitability of transduced VTA-projecting ACC neurons. Scale bars = 25 mV, 100 ms. **(F)** Summary graph showing the volume of solution consumed between groups during overnight conditioning sessions (ON1-6) [*F*_(15,140)_ = 36.52, *p* < 0.0001, two-way repeated-measures ANOVA with Bonferroni *post hoc* test] (DIO-mCherry-sacch: *n* = 7; DIO-mCherry-Oxy: *n* = 8; DIO-hM4di-sacch: *n* = 8; DIO-hM4di-Oxy: *n* = 9]. **(G)** Summary graph showing the locomotor activity counts across conditioning sessions [*F*_(15,140)_ = 10.36, *p* < 0.0001, two-way repeated-measures ANOVA with Bonferroni *post hoc* test] (DIO-mCherry-sacch: *n* = 7; DIO-mCherry-Oxy: *n* = 8; DIO-hM4di-sacch: *n* = 8; DIO-hM4di-Oxy: *n* = 9). **(H)** Summary graph showing the withdrawal score of control and oxycodone-conditioned mice expressing mCherry or hM4di in ACC neurons that project to the VTA. Experiments were performed in the presence of CNO (2 mg/kg, i.p.) [*F*_(3,28)_ = 327.4, *p* < 0.0001, one-way ANOVA with Bonferroni *post hoc* test] (DIO-mCherry-sacch: *n* = 7; DIO-mCherry-Oxy: *n* = 8; DIO-hM4di-sacch: *n* = 8; DIO-hM4di-Oxy: *n* = 9). **(I)** Summary graph showing that inhibition of VTA-projecting ACC neurons blocks oxycodone-induced CPP. Experiments were performed in the presence of CNO (2 mg/kg, i.p.) [*F*_(3,28)_ = 7.27, *p* = 0.0009, one-way ANOVA with Bonferroni *post hoc* test] (DIO-mCherry-sacch: *n* = 7; DIO-mCherry-Oxy: *n* = 8; DIO-hM4di-sacch: *n* = 8; DIO-hM4di-Oxy: *n* = 9). **(J)** Summary graph showing the mLs of water consumed in the unpaired and paired chambers by control and oxycodone-conditioned mice injected with DIO-mCherry or DIO-hM4di in the ACC. Experiments were performed in the presence of CNO (2 mg/kg, i.p.) [*F*_(3,58)_ = 3.264, *p* = 0.0277, two-way ANOVA with Bonferroni *post hoc* test] (DIO-mCherry-sacch: *n* = 7; DIO-mCherry-Oxy: *n* = 8; DIO-hM4di-sacch: *n* = 8; DIO-hM4di-Oxy: *n* = 9). *p < 0.05, ^**^p < 0.001, ^***^p < 0.0001. RMP, resting membrane potential.

In order to selectively target this pathway, we utilized a Cre-dependent chemogenetic approach. Prior to the onset of behavioral experiments, mice were injected with a retrograde viral construct containing Cre-recombinase into the VTA (Figure 4A). Four weeks later, mice were injected with either a Cre-dependent mCherry (vehicle control; DIO-mCherry) or a Cre-dependent hM4di(Gi) inhibitory virus conjugated to the mCherry fluorophore (DIO-hM4di). This permitted mCherry or hM4di(Gi) expression on ACC neurons that project to the VTA (VTA-projecting ACC neurons) (Figure 4B). Following a four-week viral incubation period, mice underwent oral-SA-CPP training (Figure 4A).

First, we wanted to investigate whether VTA-projecting ACC neurons expressed increases in IME following re-exposure to the drug-paired chamber, similar to what we observed previously (Figure 3). To do this, we performed whole-cell *ex vivo* electrophysiology on a subset of DIO-mCherry mice that underwent saccharin or oxycodone (0.5 mg/ml) oral-SA-CPP. Immediately following the extinction test, brain slices containing the ACC were obtained. We observed that ACC neurons that project to the VTA had similar morphological and electrophysiological characteristics compared to randomly sampled L5 thick-tufted pyramidal neurons targeted previously, albeit with significant differences in capacitance and resistance (Table 2). Additionally, we observed that these fluorescently-labeled VTA-projecting ACC neurons predominantly were located in L5 of the ACC (Figure 4B). When measuring the IME of VTA-projecting ACC neurons, we found a significant increase in the IME in DIO-mCherry oxycodone-trained mice compared to DIO-mCherry saccharin controls (*F*_(14,336)_ = 2.551, *p* = 0.0017, two-way repeated-measures ANOVA with Bonferroni *post hoc* test; Figure 4C). This result was a similar to what we observed when measuring the IME of randomly sampled L5 ACC thick-tufted pyramidal neurons (Figure 3).

**TABLE 2:** Electrophysiological properties of VTA-projecting ACC neurons.

Properties	L5 ACC-thick-tufted PyNs	VTA projecting ACC neurons	*p* value
RMP	−73.22 ± 0.68	−74.18 ± 0.84	0.3735
Capacitance	149.8 ± 5.4	121.9 ± 5.4	0.0006***
Resistance	120.2 ± 5.9	195.4 ± 18.6	0.0002***
SAG	1.929 ± 0.13	1.973 ± 0.27	0.8824

p Value calculated using a Student’s unpaired t-test. ***p < 0.001.

RMP=resting membrane potential

We next aimed to test whether inhibiting VTA-projecting ACC neurons influenced context-induced drug seeking behaviors. For these experiments, mice expressing Cre-dependent hM4di in VTA-projecting ACC neurons underwent oral-SA-CPP training as this approach allowed us to inhibit VTA-projecting ACC neurons upon activation with the actuator CNO (Figures 4D,E). As observed previously (Figures 1, 2), during conditioning, we found that mice who self-administered oxycodone (0.5 mg/ml) increased their consumption of oxycodone during each overnight session in the paired chamber (ON 2, 4, and 6) (DIO-mCherry-Oxy: ON2 vs. ON6: *p* = 0.0441; DIO-hM4di-Oxy: ON2 vs. ON6: *p* = 0.0396; two-way repeated measures ANOVA with Bonferroni *post hoc* test) (Figure 4F) and, by ON 6, these mice had increased locomotor activity compared to controls (*F*_(15,140)_ = 10.36, p < 0.0001; two-way repeated measures ANOVA with Bonferroni *post hoc* test) (Figure 4G). These results suggest that oxycodone self-administering mice were opioid dependent, which was supported by the significant increases in the withdrawal score in oxycodone self-administering mice injected with the DIO-mCherry (DIO-mCherry-Oxy) compared to saccharin controls when CNO (2 mg/kg, i.p.) was administered 15 min prior to the withdrawal test (vs. DIO-mCherry-sacch: *p* < 0.0001; vs. DIO-hM4di-sacch: *p* < 0.0001; one-way ANOVA with Bonferroni *post hoc* test) (Figure 4H). However, spontaneous opioid withdrawal was completely blocked by inhibition of VTA-projecting ACC neurons (DIO-hM4di-Oxy) in the presence of CNO (2 mg/kg, i.p.) (vs. DIO-mCherry-sacch: *p* > 0.999; vs. DIO-hM4di-sacch: *p* > 0.9999; one-way ANOVA with Bonferroni *post hoc* test) (Figure 4H).

One day following the withdrawal tests (48 h following the last oral-SA-CPP conditioning session), at a time point when CNO levels are significantly reduced (Jendryka et al., 2019), mice received a second injection of CNO (2 mg/kg) 15 min prior to the CPP and 1 h extinction test. As expected, we found that DIO-mCherry-Oxy mice had a significant preference for the oxycodone-paired chamber compared to DIO-mCherry-sacch controls when CNO (2 mg/kg, i.p.) was administered 15 min prior to the extinction test (*p* = 0.010; one-way ANOVA with Bonferroni *post hoc* test) (Figure 4I). However, when VTA-projecting ACC neurons were inhibited *via* CNO injection (2 mg/kg, i.p.), we found that oxycodone-induced CPP was blocked, as there was no significant difference between DIO-hM4di-Oxy and DIO-hM4di-sacch groups in the amount of time spent in the paired chamber (*p* > 0.9999; one-way ANOVA Bonferroni *post hoc* test), with a significant decrease in the CPP score in DIO-hM4di-Oxy compared to DIO-mCherry-Oxy mice (*p* = 0.0261; one-way ANOVA Bonferroni *post hoc* test) (Figure 4I).

Lastly, we monitored the volume of water consumed in each chamber during the 1 h extinction test and found that DIO-mCherry-Oxy mice engaged in drug-seeking as measured by significantly more water consumed in the paired side compared to DIO-mCherry-sacch (*p* = 0.0124; two-way ANOVA with Bonferroni *post hoc* test) (Figure 4J). Furthermore, we found that inhibiting VTA-projecting ACC neurons prevented oxycodone-induced drug-seeking, as DIO-hM4di-Oxy mice had no difference in consumption of water in the paired chamber compared to DIO-hM4di-sacch controls (*p* = 0.5332; two-way ANOVA with Bonferroni *post hoc* test) (Figure 4J).

## Discussion

Here, using a preclinical model capable of measuring context-induced drug-seeking behavior, we show that inhibition of the ACC and its projections to the VTA, block context-induced drug seeking as well as spontaneous opioid withdrawal. Additionally, we show that VTA-projecting ACC neurons express increases in their IME following oxycodone self-administration, a time point corresponding to the behavioral phenotypes of drug seeking and spontaneous opioid withdrawal. Overall, these results provide evidence that the ACC is directly involved in context-induced drug seeking and opioid withdrawal and that this may be regulated by a specific class of ACC projecting neurons.

We employed our novel method that allows mice to orally self-administer an opioid, oxycodone, and 0.2% saccharin, a substance that does not evoke CPP (White and Carr, 1985; Stefurak and van der Kooy, 1992; Agmo and Marroquin, 1997; McKendrick et al., 2020b), each within a specific context of a three-compartment behavioral apparatus. Following the self-administration paradigm, we were able to then measure choice behaviors associated with place preference and context-induced drug seeking as defined by the amount of water consumed in contexts associated with drug (oxycodone) and non-drug (0.2% saccharin) during an extinction test.

As we have observed, this method has advantages that extrapolate to the clinical manifestations of OUD. Namely, during the long-access self-administration sessions, when mice were given access to opioids, we observed an escalation of opioid intake over the three overnight sessions, which is commonly observed when implementing long-access self-administration paradigms in preclinical models (Edwards and Koob, 2013; Blackwood et al., 2019). This may be attributed to neuroadaptive dysregulations that result in increased hedonic or reward set points (Weiss et al., 2001) and/or to opioid tolerance. Tolerance is a common physiological symptom associated with subjects who misuse opioids, along with physical dependence to opioids that can manifest as somatic signs of withdrawal (Miller and Greenfeld, 2004; American Psychiatric Association, 2013). In our behavioral model, we observed that mice who self-administer oxycodone at doses greater than or equal to 0.5 mg/ml, expressed somatic signs of withdrawal during abstinence, indicative of the physical dependence to opioids. Additionally, we found that oxycodone self-administering mice consumed less 0.2% saccharin solution over the duration of training paradigm when oxycodone was not available. This behavior is likely caused by the physical signs of withdrawal that cause mice to be less receptive toward non-rewarding substances. In line with this, we observed that a significant increase in the time spent on the paired chamber was only observed for the middle dose group, whereas a significant decrease in the time spent on the unpaired chamber was only observed for the high dose group. Additionally, only the middle dose group consumed significantly more water in the oxycodone-paired chamber during the extinction test. These results are potentially due to differences in withdrawal phenotypes between middle and high dose groups. First, we observed a significant decrease in activity between ON 3 versus ON 5 (when 0.2% saccharin was available) in the high dose group, but not in the middle dose group. This suggests that the high dose group was potentially expressing withdrawal phenotypes at earlier time points compared to the middle dose group. We speculate that the high dose group was undergoing withdrawal by ON 5, potentially resulting in the avoidance for the unpaired chamber observed during the CPP test. Furthermore, we observed that the high dose group expressed a significant decrease in locomotor activity compared to the middle dose group 24 h following the last conditioning session, potentially explaining why only the middle dose group consumed significantly more water in the oxycodone-paired chamber during the extinction test.

Interestingly, we found that global inhibition of the ACC attenuated spontaneous opioid withdrawal in oxycodone self-administering mice, which is in line with our previous finding that showed that global inhibition of the ACC attenuated morphine-induced spontaneous opioid withdrawal (McDevitt et al., 2021). The results of both studies are very similar despite some key differences in the drug used (oxycodone vs. morphine), route of administration (self-administered vs. intraperitoneal injection), and drug exposure paradigm (alternating overnight sessions during 6 overnights vs. twice a day escalating bolus dose). Oxycodone and morphine belong to the same chemical class, phenanthrenes, which may impart similar alterations to opioid receptor function within the central nervous system, potentially explaining the comparable attenuation of spontaneous opioid withdrawal following global ACC inhibition. However, multiple differences exist between oxycodone and morphine, including receptor binding affinity for μ and kappa opioid receptors (Chen et al., 1991; Nielsen et al., 2007), pharmacokinetic profiles (blood brain barrier penetration; metabolism, elimination, and bioavailability) (Hoskin et al., 1989; Mandema et al., 1996; Takala et al., 1997; Coffman et al., 1998; Wandel et al., 2002; Lalovic et al., 2004; Boström et al., 2006), and effects on anti-nociception (Kalso, 2005; Lenz et al., 2009; Drewes et al., 2013; Guo et al., 2018). Based on these differences, it is tempting to speculate that the somatic signs of opioid withdrawal may share a common neurocircuit pathway through the ACC, but future studies are required to investigate the effects of ACC inhibition on the physical signs of opioid withdrawal following administration of other classes of opioids, including benzomorphans, phenylpiperidines (e.g., fentanyl), and diphenylheptanes (e.g., methadone).

Although we did not test the effects of different classes of opioids, we were able to show that inhibition of a specific subclass of ACC projection neurons (i.e., VTA projecting ACC neurons that were hyperexcitable following context re-exposure) blocked spontaneous opioid withdrawal following oxycodone self-administration. Specifically inhibiting ACC projections to the VTA would be expected to reduce ACC-mediated glutamate transmission on dopamine neurons in the VTA. This decrease in glutamate release in the VTA would be expected to reduce dopamine neuron excitability, subsequently leading to depleted dopamine release in VTA projecting brain regions. This depletion of dopamine may be beneficial for reducing somatic signs of opioid withdrawal as it has been shown preclinically that activation of a specific class of dopamine receptor, the D_2_ dopamine receptor, increases the severity of withdrawal symptoms, while blocking the D_2_ dopamine receptor reduces withdrawal symptoms (Cox et al., 1976; El-Kadi and Sharif, 1998; Dunn et al., 2019). Interestingly, increasing dopamine levels with the dopamine precursor L-DOPA, which would be expected to target all dopamine receptors with a lack of specificity for specific dopamine receptor subtypes, dose dependently increases several symptoms of opioid withdrawal, while decreasing others (Herz et al., 1974; El-Kadi and Sharif, 1998; Dunn et al., 2019). Together, these results suggest that targeting specific dopamine receptors may provide optimal prevention of opioid withdrawal. Here, we observed complete block of spontaneous opioid withdrawal through direct inhibition of ACC to VTA connections, which is in contrast to the attenuation of spontaneous opioid withdrawal that was observed following global ACC inhibition. Potentially then, precise targeting of neurocircuit pathways, similar to precise targeting of receptor pathways in pharmacology, may provide optimal prevention of opioid withdrawal. Treatments using brain stimulation pathways have been approved by the FDA for treating opioid withdrawal (Administration, 2017), potentially paving the way for future innovative and neurocircuit-specific electrical stimulation therapy.

### Anterior cingulate cortex in drug-context associations and drug seeking

Pavlovian learning, the ability to form relationships between temporally-associated stimuli, is critically important for context-induced relapse where re-exposure to drug-associated contexts evokes strong drug-craving (O’Brien and Ternes, 1986; O’Brien et al., 1992). Understanding which brain regions are involved in encoding context associations is important because drug-context associations are a required precursor for context-induced drug seeking. Here, we discovered that global inhibition of the ACC as well as inhibition of VTA-projecting ACC neurons attenuates and blocks oxycodone-induced CPP, respectively. This important finding contributes to our understanding of brain regions that are involved in opioid-context associations as well as helps build a neurocircuit map of interconnected brain regions that function to regulate opioid-induced Pavlovian learning. For example, evidence suggests that the amygdala (Zarrindast et al., 2003; Rezayof et al., 2007), hippocampus (Rezayof et al., 2003; Rezayof et al., 2006; Portugal et al., 2014; Li et al., 2017; Nam et al., 2019), somatosensory cortex (Meng et al., 2009), and VTA (Harris et al., 2004; Ma et al., 2009; Moaddab et al., 2009; Narita et al., 2010; Esmaeili et al., 2012; Koo et al., 2012) are important brain structures capable of modulating opioid-induced CPP (McKendrick and Graziane, 2020). The ACC receives or sends projections to the amygdala, hippocampus, somatosensory cortex, and VTA (Gabbott et al., 2005; Fillinger et al., 2017, 2018), potentially acting as an important structure involved in the simultaneous transmission of several signals within this neurocircuit connection. It is difficult to determine whether these signals are related to reward, aversion, anticipatory choice behaviors, motivation, and/or any other factors that are involved in drug-seeking behaviors.

Evidence suggests that global inhibition of the ACC as well as inhibition of VTA-projecting ACC neurons evokes CPP in models of chronic inflammatory pain and chronic constriction injury (CCI), respectively (Sukjae Joshua et al., 2017; Gao et al., 2020). Additionally, it has been shown that VTA-projecting ACC neurons are hyperexcitable in a model of CCI (Gao et al., 2020). These findings suggest that hyperexcitability of the VTA-projecting ACC neurons is associated with an aversive state and that inhibition of the ACC or ACC projections to the VTA reduce aversive states, leading to CPP evoked by negative reinforcement. Our results are in line with these findings. First, we found that following oxycodone self-administration, at a time point when mice expressed spontaneous opioid withdrawal, VTA-projecting ACC neurons were hyperexcitable. Additionally, we found that inhibition of these neurons was sufficient to block oxycodone-induced CPP and drug-seeking behaviors. Seminal work by Shaham et al. showed that spontaneous opioid withdrawal reinstates heroin seeking (Shaham et al., 1996). Potentially then, inhibition of the VTA-projecting ACC neurons is able to reduce aversive states associated with the somatic or psychological signs of opioid withdrawal. Doing so, mitigates the need to seek drug in order to avoid negative affective states and maintain a hedonic homeostatic balance.

Others posit that the ACC to VTA neurocircuit connection is crucial for the behaviors elicited by anticipatory reward processing and motivation (Elston and Bilkey, 2017; Elston et al., 2019). It was shown that in the absence of a barrier, when animals approach a zone known to contain a reward, there are increases in ACC and VTA theta power and local field potential coherence, which reflects phase consistency between ACC and VTA regions that communicate in the theta band (4–12 Hz) (Elston et al., 2019). Given this, it is plausible that inhibition of the VTA-projecting ACC neurons disrupts the motivation and/or approach behavior to seek reward in the context paired with oxycodone.

### Thick- and thin-tufted pyramidal neurons in the anterior cingulate cortex and behavioral correlates

We observed increases in the IME on L5 thick-tufted PyNs in the ACC following context-re-exposure in oxycodone oral self-administering male mice. These results are in line with other studies that have shown similar neuroadaptations in male mice following intravenous self-administration of remifentanil (Anderson et al., 2021), demonstrating potentially shared neuroadaptations between different opioid drugs and preclinical models of self-administration. Additionally, we observed a similar enhancement in the IME of VTA-projecting ACC PyNs, which suggests that this subpopulation expresses enhanced sensitivity to incoming stimuli. Given that ACC PyNs directly target dopamine neurons (Overton et al., 1996; Tong et al., 1996), reliable signal transfer from the ACC to the VTA may provide robust dopamine signaling throughout VTA-projecting brain regions, thereby promoting behaviors linked to drug seeking during abstinence, but not important for opioid reinforcement (Pettit et al., 1984).

Furthermore, our observed increases in L5 thick-tufted PyNs excitability in the ACC during a time point corresponding to opioid withdrawal may reflect neuroadaptations evoked by dysregulation of brain stress systems. Recent evidence suggests that brain stress systems drive hyperkatifeia, allostasis and drug intake (Koob and Schulkin, 2019; Koob, 2020) with the amygdala acting as a key hub in this process (Carmack et al., 2022). L5 thick-tufted PyNs make reciprocal excitatory glutamatergic synaptic connections with the amygdala (Etkin et al., 2011; Cheriyan et al., 2016; Burgos-Robles et al., 2017; Rolls, 2019). Potentially then, this neurocircuit connection between L5 thick-tufted PyNs and the amygdala may also contribute to our observed increases in context-induced drug seeking during 24 h abstinence from oxycodone self-administration.

Lastly, L2/3 and L5 thin-tufted PyNs in the ACC receive long-range and local inputs similar to L5 thick-tufted PyNs in the ACC, while also providing local excitation on L5 thick-tufted PyNs (Little and Carter, 2013; Collins et al., 2018; Anastasiades et al., 2021). Based on this, it is speculated that thin-tufted PyNs may amplify incoming signals on thick-tufted PyNs, ensuring reliable output to subcortical structures (Anastasiades and Carter, 2021). Our results suggest that although L5 thin-tufted PyNs did not express enhanced IME, they did demonstrate a significant reduction in the rheobase, the minimum amount of current required to evoke and action potential, in mice undergoing context re-exposure during 24 h abstinence from oxycodone self-administration. Therefore, it is possible that L5 thin-tufted PyNs in the ACC also undergo oxycodone-induced changes in their intrinsic properties that enable them to contribute to increased excitability within the ACC of oxycodone exposed mice. Importantly, the ACC is made up of multiple layers (I, II/III, V, and VI) each with distinct populations of PyNs and interneurons. More work is required to identify how each of these subpopulations of neurons is altered in the ACC following drug exposure both *in vivo* and *ex vivo* and whether these potential alterations contribute to context-induced relapse and opioid withdrawal.

## Limitations

Our results are not without limitations. First, extra-telencephalic pyramidal neurons in the ACC send axons that are multi-projectional, thus sending branches to more than one subcortical structure (Kita and Kita, 2012; Shepherd, 2013; Anastasiades and Carter, 2021). Given this, our approach used to inhibit ACC pyramidal neurons that project to the VTA is also likely inhibiting inputs to other subcortical structures, including the striatum, periaqueductal gray, thalamus, brainstem, and spinal cord (Fillinger et al., 2017, 2018). Each of these brain regions have been shown to be involved in motivated behaviors and/or withdrawal (Wei et al., 1972; Tremblay and Charton, 1981; Maldonado et al., 1992; Delgado et al., 2003; Palmiter, 2008; Silva and McNaughton, 2019; Iglesias and Flagel, 2021). Because of this, when the tools are available, it will be important to decipher whether specific pyramidal neurons or their specific inputs to individual brain regions are important for regulating the behaviors that we tested here.

Second, our extinction test which was used to measure context-induced drug seeking occurred within a timeframe when mice were undergoing spontaneous opioid withdrawal. Therefore, it is difficult to interpret whether ACC inhibition attenuates withdrawal or whether ACC inhibition directly blocks context-associations. Importantly, clinically, reducing negative affective states is likely to diminish the motivation to seek drugs (Solomon and Corbit, 1973). Evidence suggests that ACC activation is closely linked to negative affective states, including pain processing (van Heukelum et al., 2020). Therefore, it is plausible that ACC activation following re-exposure to drug associated cues stimulates brain regions involved in negative affective states, thus driving drug craving. Future studies are required to identify the exact role of the ACC in these behaviors.

Third, we acknowledge that our approach did not include female mice thereby limiting comparisons between sexes. Based on previously published findings, sex differences have been observed in the acquisition of self-administered oxycodone, fentanyl, and heroin (Lynch and Carroll, 1999; Townsend et al., 2019; Fulenwider et al., 2020) and tolerance and dependence to morphine (Craft et al., 1999). Additionally, it has been widely shown that opioids modulate brain regions differentially based on sex (Harte-Hargrove et al., 2015; Doyle et al., 2017; Ryan et al., 2018; Enman et al., 2019; Luster et al., 2020; Anderson et al., 2021). Clinically, the proportion of female admissions reporting primary misuse of oxycodone is nearly three times that of men (7.2% vs. 2.8%) (Administration, 2014), and substance use disorders involving opioid analgesics are thought to develop more rapidly in women (Medicine, 2016). Based on this, future preclinical investigations will need to focus on sex comparisons related to the volume of oxycodone consumption during self-administration, oxycodone-induced CPP, context-induced drug seeking, and spontaneous opioid withdrawal.

## Conclusion and future directions

Our studies demonstrate the important function of the ACC in many key aspects of OUDs. Building upon these findings, it will be important to investigate (i) the role of the ACC following longer durations of oxycodone exposure in our model (e.g., 7 vs. 3 ON sessions), (ii) the role of the ACC following re-exposure to specific contexts at time points falling outside time points associated with somatic signs of opioid withdrawal, and (iii) ACC dynamics during re-exposure to the drug-paired and unpaired side using *in vivo* imaging approaches. These important future directions will likely lead to a more complete understanding of drug-induced adaptations alter CNS function subsequently resulting in the criteria used to diagnose OUD.

## Data availability statement

The raw data supporting the conclusions of this article will be made available by the authors, without undue reservation.

## Ethics statement

The animal study was reviewed and approved by Pennsylvania State University College of Medicine Institutional Animal Care and Use Committee.

## Author contributions

GM and NG designed the experiments. GM, DM, AC, PS, and NG collected data and performed the analyses. GM and NG wrote the manuscript. All authors contributed to the article and approved the submitted version.
